# Supplementary material for: A novel serum miRNA-pair classifier for diagnosis of sarcoma
Source: PLoS One. 2020 Jul 16;15(7):e0236097. doi: 10.1371/journal.pone.0236097 (PMC7365454; doi:10.1371/journal.pone.0236097)
Supplement: S1 Table — (DOCX) [file pone.0236097.s001.docx]

| Dataset ID | Sample ID | Histological | Metastatic |
| --- | --- | --- | --- |
| E-MTAB-3273 | Sample_1 | Synovial sarcoma | Localized |
| E-MTAB-3273 | Sample_2 | Synovial sarcoma | Metastatic |
| E-MTAB-3273 | Sample_3 | Synovial sarcoma | Metastatic |
| E-MTAB-3273 | Sample_4 | Synovial sarcoma | Metastatic |
| E-MTAB-3273 | Sample_5 | Synovial sarcoma | Metastatic |
| E-MTAB-3273 | Sample_6 | Healthy | No disease |
| E-MTAB-3273 | Sample_7 | Healthy | No disease |
| E-MTAB-3273 | Sample_8 | Healthy | No disease |
| E-MTAB-3273 | Sample_9 | Healthy | No disease |
| E-MTAB-3273 | Sample_10 | Healthy | No disease |
| E-MTAB-3888 | Synovial_Sarcoma_1 | Synovial sarcoma | NA |
| E-MTAB-3888 | Synovial_Sarcoma_2 | Synovial sarcoma | NA |
| E-MTAB-3888 | Synovial_Sarcoma_3 | Synovial sarcoma | NA |
| E-MTAB-3888 | Synovial_Sarcoma_4 | Synovial sarcoma | NA |
| E-MTAB-3888 | Synovial_Sarcoma__5 | Synovial sarcoma | NA |
| E-MTAB-3888 | Healthy_Donor__1 | Healthy | No disease |
| E-MTAB-3888 | Healthy_Donor__2 | Healthy | No disease |
| E-MTAB-3888 | Healthy_Donor__3 | Healthy | No disease |
| E-MTAB-3888 | Healthy_Donor__4 | Healthy | No disease |
| E-MTAB-3888 | Healthy_Donor__5 | Healthy | No disease |
| E-MTAB-3888 | Leiomyosarcoma__1 | Leiomyosarcoma | NA |
| E-MTAB-3888 | Leiomyosarcoma__2 | Leiomyosarcoma | NA |
| E-MTAB-3888 | Leiomyosarcoma__3 | Leiomyosarcoma | NA |
| E-MTAB-3888 | Leiomyosarcoma__4 | Leiomyosarcoma | NA |
| E-MTAB-3888 | Leiomyosarcoma__5 | Leiomyosarcoma | NA |
| E-MTAB-5126 | Healthy_control_1 | Healthy | No disease |
| E-MTAB-5126 | Healthy_control_2 | Healthy | No disease |
| E-MTAB-5126 | Healthy_control_3 | Healthy | No disease |
| E-MTAB-5126 | Healthy_control_4 | Healthy | No disease |
| E-MTAB-5126 | Liposarcoma_1 | Liposarcoma | NA |
| E-MTAB-5126 | Liposarcoma_2 | Liposarcoma | NA |
| E-MTAB-5126 | Liposarcoma_3 | Liposarcoma | NA |
| E-MTAB-5126 | Liposarcoma_4 | Liposarcoma | NA |
| E-MTAB-5126 | Liposarcoma_5 | Liposarcoma | NA |
| E-MTAB-5126 | Liposarcoma_6 | Liposarcoma | NA |

S1 Table. Clinical information of merged external dataset
