# Supplementary material for: A novel serum miRNA-pair classifier for diagnosis of sarcoma
Source: PLoS One. 2020 Jul 16;15(7):e0236097. doi: 10.1371/journal.pone.0236097 (PMC7365454; doi:10.1371/journal.pone.0236097)
Supplement: S2 Table — (DOCX) [file pone.0236097.s002.docx]

S2 Table. Nodes of the miRNA-mRNA network

| Label | Type |
| --- | --- |
| hsa-mir-518a-3p | miRNA |
| hsa-mir-571 | miRNA |
| hsa-mir-454-5p | miRNA |
| hsa-mir-378c | miRNA |
| hsa-mir-499b-3p | miRNA |
| ACVR1B | mRNA |
| CCR6 | mRNA |
| HOXC8 | mRNA |
| RPS6KA3 | mRNA |
| STK4 | mRNA |
| TYRO3 | mRNA |
| RBM8A | mRNA |
| ZNF281 | mRNA |
| ARIH1 | mRNA |
| C9orf3 | mRNA |
| PGM2L1 | mRNA |
| ZC3H12D | mRNA |
| CCNF | mRNA |
| CLU | mRNA |
| CREBBP | mRNA |
| GPR183 | mRNA |
| CELSR3 | mRNA |
| EREG | mRNA |
| G6PD | mRNA |
| GLUL | mRNA |
| HMGB1 | mRNA |
| HSPA6 | mRNA |
| MDM2 | mRNA |
| MEF2D | mRNA |
| MEN1 | mRNA |
| CIITA | mRNA |
| NDUFB5 | mRNA |
| PIP4K2A | mRNA |
| RAB5B | mRNA |
| RAD51 | mRNA |
| SNX1 | mRNA |
| TUBB2A | mRNA |
| UBE2D1 | mRNA |
| YY1 | mRNA |
| SF1 | mRNA |
| NCOA3 | mRNA |
| HIST1H2AI | mRNA |
| STC2 | mRNA |
| PER2 | mRNA |
| TIAF1 | mRNA |
| MSC | mRNA |
| NREP | mRNA |
| GTF3C4 | mRNA |
| GLP2R | mRNA |
| ADAMTS4 | mRNA |
| PUM1 | mRNA |
| USP6NL | mRNA |
| SECISBP2L | mRNA |
| RABEPK | mRNA |
| LBX1 | mRNA |
| ZNF460 | mRNA |
| TMED2 | mRNA |
| ZWINT | mRNA |
| FICD | mRNA |
| ZNF652 | mRNA |
| FBXL7 | mRNA |
| ESYT1 | mRNA |
| SUZ12 | mRNA |
| TMEM2 | mRNA |
| FKBP8 | mRNA |
| CNTNAP2 | mRNA |
| UBE2S | mRNA |
| TOR1B | mRNA |
| MRPS18B | mRNA |
| VPS28 | mRNA |
| HES2 | mRNA |
| CHTF8 | mRNA |
| PNPO | mRNA |
| AMBRA1 | mRNA |
| PMEPA1 | mRNA |
| NUFIP2 | mRNA |
| MARCH4 | mRNA |
| CXorf56 | mRNA |
| CLSPN | mRNA |
| NKAIN1 | mRNA |
| KIAA0319L | mRNA |
| ORAI2 | mRNA |
| COL18A1 | mRNA |
| SETD7 | mRNA |
| SYDE2 | mRNA |
| FIBCD1 | mRNA |
| FAM9C | mRNA |
| SLC36A1 | mRNA |
| AFG1L | mRNA |
| HSD11B1L | mRNA |
| NSUN4 | mRNA |
| SAMD12 | mRNA |
| AK2 | mRNA |
| ALDOA | mRNA |
| ALDOC | mRNA |
| ANG | mRNA |
| ARSB | mRNA |
| ASGR1 | mRNA |
| RCAN1 | mRNA |
| EFNA5 | mRNA |
| FRK | mRNA |
| HOXD9 | mRNA |
| HSF2 | mRNA |
| IL5 | mRNA |
| KCNJ10 | mRNA |
| LTBR | mRNA |
| DDR2 | mRNA |
| MAPK1 | mRNA |
| PTK6 | mRNA |
| RAP1B | mRNA |
| ATXN1 | mRNA |
| CCL16 | mRNA |
| SNRPD3 | mRNA |
| TMPO | mRNA |
| WEE1 | mRNA |
| CNBP | mRNA |
| AP3B2 | mRNA |
| MINPP1 | mRNA |
| PAN2 | mRNA |
| TRIM10 | mRNA |
| ARL4C | mRNA |
| KLF2 | mRNA |
| DDX17 | mRNA |
| NUDT21 | mRNA |
| DUSP10 | mRNA |
| CEP152 | mRNA |
| GIGYF2 | mRNA |
| LSM14A | mRNA |
| USP25 | mRNA |
| UCHL5 | mRNA |
| KDM3B | mRNA |
| IPO9 | mRNA |
| LMBR1L | mRNA |
| RNF20 | mRNA |
| SERTAD4 | mRNA |
| CRAMP1 | mRNA |
| PLEKHA1 | mRNA |
| PHACTR4 | mRNA |
| ZSCAN16 | mRNA |
| TNKS2 | mRNA |
| TANGO2 | mRNA |
| LDHD | mRNA |
| UBN2 | mRNA |
| BCLAF3 | mRNA |
| RABL3 | mRNA |
| ZNF660 | mRNA |
| FOXL2NB | mRNA |
| MINOS1 | mRNA |
| BCL2L15 | mRNA |
| SHISA9 | mRNA |
| ACACA | mRNA |
| AKT1 | mRNA |
| MYRF | mRNA |
| NDST1 | mRNA |
| KCNJ6 | mRNA |
| P4HB | mRNA |
| REST | mRNA |
| RPS7 | mRNA |
| TGFB2 | mRNA |
| TXNL1 | mRNA |
| IGDCC3 | mRNA |
| WTAP | mRNA |
| HIPK3 | mRNA |
| SEC31A | mRNA |
| ZNF609 | mRNA |
| PRKD2 | mRNA |
| RDH11 | mRNA |
| LGSN | mRNA |
| CAB39 | mRNA |
| SPA17 | mRNA |
| ARL8B | mRNA |
| WDR33 | mRNA |
| RCC2 | mRNA |
| CELF4 | mRNA |
| CYP20A1 | mRNA |
| ESYT2 | mRNA |
| CACNG8 | mRNA |
| DCTPP1 | mRNA |
| HAUS3 | mRNA |
| VPS37B | mRNA |
| KDM8 | mRNA |
| LMNB2 | mRNA |
| ANO4 | mRNA |
| PPARGC1B | mRNA |
| DPY19L3 | mRNA |
| ARGFX | mRNA |
| BMPR1A | mRNA |
| CACNA1B | mRNA |
| CSTF1 | mRNA |
| DNAH8 | mRNA |
| DNAH9 | mRNA |
| DR1 | mRNA |
| TSC22D3 | mRNA |
| ELK4 | mRNA |
| FOXO1 | mRNA |
| GLO1 | mRNA |
| IGFBP4 | mRNA |
| CXCL8 | mRNA |
| ABLIM1 | mRNA |
| MAF | mRNA |
| MDM4 | mRNA |
| MYO1D | mRNA |
| PTGFRN | mRNA |
| PTMA | mRNA |
| STYX | mRNA |
| TEF | mRNA |
| UQCRB | mRNA |
| TTF2 | mRNA |
| RNMT | mRNA |
| NAPG | mRNA |
| N4BP1 | mRNA |
| DAZAP2 | mRNA |
| MAMLD1 | mRNA |
| FEM1B | mRNA |
| SRRM1 | mRNA |
| TXNIP | mRNA |
| ACOT2 | mRNA |
| CD93 | mRNA |
| PDS5A | mRNA |
| TBC1D22A | mRNA |
| EPC2 | mRNA |
| BMP10 | mRNA |
| UBN1 | mRNA |
| NT5C3A | mRNA |
| FXYD6 | mRNA |
| PRR13 | mRNA |
| FIGN | mRNA |
| ETNK1 | mRNA |
| MAML3 | mRNA |
| VPS35 | mRNA |
| RARS2 | mRNA |
| PLEKHG5 | mRNA |
| CAPRIN2 | mRNA |
| WDR76 | mRNA |
| KLHL15 | mRNA |
| QRFPR | mRNA |
| LIN54 | mRNA |
| SRXN1 | mRNA |
| BTLA | mRNA |
| SPTSSA | mRNA |
| PDE12 | mRNA |
| PROSER2 | mRNA |
| TRIM59 | mRNA |
| NAT8L | mRNA |
| DCAF12L2 | mRNA |
| PGAM4 | mRNA |
| POM121C | mRNA |
