# Supplementary material for: A novel serum miRNA-pair classifier for diagnosis of sarcoma
Source: PLoS One. 2020 Jul 16;15(7):e0236097. doi: 10.1371/journal.pone.0236097 (PMC7365454; doi:10.1371/journal.pone.0236097)
Supplement: Attachment — Submitted filename: Response to Reviewers.docx [file pone.0236097.s007.docx]

1. Statistical analysis: in the original manuscript, the authors used AUC. In this version, the authors changed the analysis to accuracy. They should explain in the statistical analysis how they define accuracy. They also should include that they are going to describe sensitivity, specificity, negative and positive predictive values in the statistical section.

Answer: Thank you for your kind suggestions. In this study, $accuracy=\frac{TP+TN}{TP+TN+FP+FN}$

(TP: True positive; TN: True negative; FP: False positive; FN: False negative), we have explained it in statistical analysis as suggested (line: 152-156) and expanded the results (line:187-193).

2. Results: characteristic datasets. In the original manuscript the authors had included intermediate and low grade tumors. As per the authors, they have excluded these from this analysis. They may want to clarify in the characteristics of datasets section that they only included high-grade lesions.

Answer: Thank you for your kind reminding. We have clarified it in the characteristics of datasets section as suggested (line: 160).
